# Improving Emotional Intelligence through Personality Development: The Effect of the Smart Phone Application based Dharma Life Program on Emotional Intelligence

**DOI:** 10.3389/fpsyg.2018.00169

**Published:** 2018-02-23

**Authors:** Latha Poonamallee, Alex M. Harrington, Manisha Nagpal, Alec Musial

**Affiliations:** ^1^Milano School of International Affairs, Management, and Urban Policy, The New School, New York, NY, United States; ^2^Dharma Life Sciences LLC, New York, NY, United States; ^3^Independent Scholar, Previously with Dharma Life Sciences LLC, New York, NY, United States

**Keywords:** emotional intelligence, leadership development, personality development, student development, neurosciences, neuroplasticity

## Abstract

Emotional intelligence is established to predict success in leadership effectiveness in various contexts and has been linked to personality factors. This paper introduces Dharma Life Program, a novel approach to improving emotional intelligence by targeting maladaptive personality traits and triggering neuroplasticity through the use of a smart-phone application and mentoring. The program uses neuroplasticity to enable users to create a more adaptive application of their maladaptive traits, thus improving their emotional intelligence. In this study 26 participants underwent the Dharma Life Program in a leadership development setting. We assessed their emotional and social intelligence before and after the Dharma Life Program intervention using the Emotional and Social Competency Inventory (ESCI). The study found a significant improvement in the lowest three competencies and a significant improvement in almost all domains for the entire sample. Our findings suggest that the completion of the Dharma Life Program has a significant positive effect on Emotional and Social Competency scores and offers a new avenue for improving emotional intelligence competencies.

## Introduction

The role of emotional and social intelligence in leadership and professional success is well-established in literature (Goleman, 1995; Boyatzis and Van Oosten, 2003; Parker et al., 2006; Riggio and Reichard, 2008). This in turn has triggered research on understanding, assessing, and measuring the competencies (Mayer and Salovey, 1997; Morand, 2001; Boyatzis and Sala, 2004) accompanied by a growing recognition that leaders are not an exception to the emotionally charged nature of organizations. Leaders influence and improve the emotional space of organizations (Humphrey et al., 2016). A leader's emotions and the expressions of their emotions influence their followers and set the tone of the organization. One of the key roles of leaders in an organizational context is to model appropriate behaviors (Humphrey et al., 2016), thereby acting as good role models to organizational members. Subordinates look up to the way their leaders function and emulate them. Therefore, a leader who is empathetic with a highly developed capacity for emotional self-regulation inspires the followers to also be calm and empathetic in the face of a crisis. The emotions of the leaders can influence the emotions of their subordinates and vice versa. For example, due to normal workplace frustrations and workload, leaders may not communicate what they want to say in the right manner. They may get irritated and frustrated and this in turn negatively affects the mood of the other employees as well. This creates a bi-directional emotional contagion, which if recognized early, can provide an opportunity to improve the workplace environment.

Many studies highlight the importance of emotional intelligence competencies in leadership (Boyatzis and Van Oosten, 2003; Harms and Crede, 2010). For example, Boyatzis and Ratti (2009) demonstrate how “outstanding” leaders had more of these competencies present, when compared to the average and above average leaders. For example, the outstanding leader took more initiatives to come up with solutions; they spent more time in planning about the future of the organization and were more assertive in their mode of communication. Similarly, Boyatzis et al. (2012) study found that social and emotional competencies such as conflict management, empathy, organizational management, and influence were positive. Further, emotionally intelligent managers are more satisfied with their work, more committed to their career (occupational commitment), and more committed to their organizations (organizational commitment) (Carmelli, 2003). Those with higher emotional intelligence are less likely to withdraw from their organization, which is crucial when trying to retain valuable employees. Higher emotional intelligence has also been associated with behaviors that support organizational goals amongst managers (Côté and Miners, 2006). Overall, therefore, we can conclude that emotional intelligence plays an important role in determining how a leader performs in an organization. However, despite the call to develop more holistic approaches to leadership development (Boyatzis et al., 2002), research on how to develop these competencies is still an evolving area that is lagging behind practice, a bulk of which is about how to use the assessment tools in executive coaching (Neale et al., 2009). Moreover, most current programs primarily focus on enhancing competencies at a behavioral level.

This paper adds to this literature by introducing the Dharma Life Program, an app based personality development tool. Grounded in the state of the art scientific findings on personality theory, trait modification theory, neuroplasticity, and cognitive behavioral modification approaches (Stiles, 2000; Galván, 2010), this tool goes one step further and is focused on developing one's personality traits at a deep, neurobiological level thereby enhancing participants' emotional and social intelligence. A growing body of literature suggests a significant correlation between EI and personality traits such as extraversion, openness, conscientiousness, and agreeableness (Ghiabi and Besharat, 2011; Nawi et al., 2012; Kappagoda, 2013). These findings indicate that a person who scores high on self-awareness, social awareness, relationship management, and self-management is likely to be outgoing, creative, and open to exploring new ideas. They also find that personality traits could effectively predict variances in EI, out of which conscientiousness appears to have the highest correlation with EI (Nawi et al., 2012). A person who scores high on conscientiousness trait can be expected to possess greater self-discipline and greater capacity for emotional self-regulation. The association of extraversion (and other positive traits) and neuroticism with EI can be justified based on the individuals' tendencies to experience positive and negative emotions, respectively. Those who score high on extraversion will have a greater tendency to experience pleasure. On the other hand, those high on neuroticism will have a greater tendency to experience negative emotions, along with poor adjustment. Based on the assumption that unbalanced personality traits affect human flourishing, this tool focuses on balancing traits identified as unbalanced in the individual so their emotional and social intelligence can be enhanced.

The paper then presents a study in which 26 leadership development participants underwent the Dharma Life Program intervention. The study finds that Dharma Life Program positively influences the emotional intelligence of participants. The paper concludes with a discussion of the findings and implications for future research and practical applications.

## Emotional intelligence

Peter Salovey and John Mayer were early adopters of the term emotional intelligence. They initially defined emotional intelligence as, “a form of intelligence that involves the ability to monitor one's own and other's feeling and emotions, to discriminate among them and to use this information to guide one's thinking and actions” (Salovey and Mayer, 1990). Eventually, based on further research, the authors refined emotional intelligence as, “the ability to perceive emotion, integrate emotion to facilitate thought, understand emotions, and to regulate emotions to promote personal growth” (Mayer and Salovey, 1997). Their model not only understands emotional intelligence as a form of intelligence, i.e., a cognitive ability, but also presents a practical step-wise succession by which a person can become emotionally intelligent. These steps are represented as four levels: *emotional perception, emotional assimilation, emotional understanding, and emotional management*. Emotional perception describes a self-awareness of accurately expressing one's emotions. The next level, emotional assimilation, distinguishes between different emotions thereby allowing the individual to identify how his/her thought process is affected. Emotional understanding applies the assimilation concept to both understand more complex emotions as well as better recognize transitions between emotions. The last level is emotional management, which involves the individual becoming adept at successfully controlling their impulses and managing their emotional reactivity in order to analyze a given situation and behave rationally.

In addition to being viewed as an ability, emotional intelligence has also been considered from a trait perspective. Analyses of trait based emotional intelligence have shown that it has a demonstratively similar factor space with the Big Five dimensions of personality (Petrides et al., 2007a). Further, a recent meta-analysis of 142 data sources examined the relationship between trait and ability emotional intelligence and the general factor of personality (GFP) (van der Linden et al., 2016). It was found that not only does GFP moderately correlate with ability emotional intelligence, it largely overlaps with trait emotional intelligence.

One of the most commonly used contemporary models for assessing emotional intelligence comes from the work of Goleman (2001a,b) who breaks down the construct into an emotional intelligence competency inventory (ECI) consisting of four clusters and twenty competencies:

1. Self-Awareness: Emotional Self-Awareness, Accurate Self-Assessment, and Self-Confidence.
2. Self-Management: Self-Control, Trustworthiness, Conscientiousness, Adaptability, Achievement Drive, and Initiative.
3. Social Awareness: Empathy, Social Orientation, and Organizational Awareness.
4. Relationship Management: Developing Others, Influence, Communication, Conflict Management, Leadership, Change Catalyst, Building Bonds, Teamwork, and Collaboration.

Subsequently, this inventory was expanded this to include social intelligence leading to the emotional and social competency inventory (ESCI), which functions with the same clusters albeit with a reduction to 12 competencies (Boyatzis, 2007):

1. Self-Awareness: Emotional Self-Awareness.
2. Self-Management: Achievement Orientation, Adaptability, Emotional Self-Control, and Positive Outlook.
3. Social Awareness: Empathy and Organizational Awareness.
4. Relationship Management: Conflict Management, Coach and Mentor, Influence, Inspirational Leadership, and Teamwork.

ESCI incorporates two dimensions of emotional intelligence: ability or competency based measures that can be captured via maximum performance tests (Mayer et al., 1999; Côté, 2014) and traits based intelligence (Petrides et al., 2007b). Ability emotional intelligence is linked with general intelligence measures, coping skills, and emotional regulation. Trait intelligence, on the other hand, highly correlates with personality traits and is essentially a constellation of emotional perceptions operationalized via questionnaires and ratings scales (Petrides et al., 2007b). Therefore, ESCI can be considered a measurement of a mixed competency model of emotional intelligence as it is partially associated with both definitions and is an appropriate tool for our purpose.

Furthermore, the ESCI has been thoroughly investigated as an effect measurement to operationalize the construct of emotional intelligence. Research has found the ESCI to be a particularly useful measure for predicting leadership effectiveness through both 360-degree and self-reported assessments. In a multi-rater, cross-sectional study design where an individual's leadership competencies are assessed through self-assessment questionnaire as well as questionnaires offered to co-workers, the ESCI predicted leaders' effectiveness in various contexts such as family businesses (Miller, 2014), public institutions such as universities (Babu, 2016), and private businesses (Kendall, 2016). ESCI has also been reliable in predicting leadership effectiveness in various professions such as knowledge workers (Mahon et al., 2014), engineers (Boyatzis et al., 2017), physicians (Quinn, 2015), financial services salesforce (Boyatzis et al., 2012), university presidents (Babu, 2016), and IT professionals and IT managers (Pittenger, 2015). There is also evidence for cross-cultural validity and utility for this inventory. For example, Bajaj and Medury (2013) conducted a study in India that predicted a manager's leadership effectiveness with the ESCI ratings of 120 subordinates in addition to being useful in transforming the leadership style of a sample of managers.

## Personality traits

Personality traits can be defined as habitual patterns of behavior, thought, and emotion with the intersection and interplay of these traits forming a “personality” (Matthews and Corr, 2009; MerriamWebster, 2017^1^). Personality traits show relative stability (Watson, 2004, p. 321), allowing for a person to form a distinct pattern of behavior that is recognizably typical and which falls across a spectrum of traits (e.g., to what extent/how typically a person engages in a pattern of behavior, thoughts, or emotional reactivity characterized within a trait). These traits reflect automatic ways in which a person interprets and reacts to their environment. Additionally, traits can become maladaptive when they become an overarching automatic response which does not “adjust adequately or appropriately to the environment or situation (Oxford Dictionary.com, 2017).” An adaptive trait on the other hand creates an automatic response that “adjusts adequately or appropriately to the environment or situation.” The Dharma Life Program, a smart-phone application based personality development program, seeks to enable individuals to develop more adaptive responses to their environments by targeting and modifying their maladaptive traits. The Dharma Life program is based on the premise that an individual can develop a maladaptive trait when they repeatedly experience external and internal stimuli that trigger maladaptive responses. These repetitions create a specific neural structure (Kimberley et al., 2010, p. 851). In order to employ the trait more appropriately, it is necessary to perform repetitions to counter both the thoughts and behaviors inherent in to the maladaptive trait, and thus modify the existing neural structure. This can be achieved by utilizing the concept of neuroplasticity.

Neuroplasticity is defined as “the brain's ability to change, remodel and reorganize, for the purpose of better adapting to new situations” (Demarin and Morović, 2014, p. 209). “New situations” can refer to a vast array of internal and external stimuli, including physical environment, social environment, a shift in attention, emotions, or thoughts. A literature review by Sharma et al. (2013) speaks to the broad span of potential applications for the concept of neuroplasticity. The researchers note that studies utilizing the concept of neuroplasticity found it effective for:

“modification in overall cognitive strategies to successfully cope with new challenges (i.e., attention, behavioral compensation) (Bury and Jones, 2002), recruitment of new/different neural networks (Johansen-Berg et al., 2002; Fridman et al., 2004; Lotze et al., 2006; Heuninckx et al., 2008), or changes in strength of such connections or specific brain areas in charge of carrying out a particular task (i.e., movement, language, vision, hearing) (Cohen et al., 1997; Grefkes et al., 2008) recruitment of new/different neural networks, or changes in strength of such connections or specific brain areas in charge of carrying out a particular task (p. 1).”

To trigger the brain to form new neural connections or alter existing neural connections to “successfully cope with new challenges” (Sharma et al., 2013, p. 1), a repetition of the modifying stimuli must take place. For example, in a study conducted by Ma et al. (2010), healthy subjects performed a specific finger movement task (a motor skill), every day for 4 weeks. A fMRI revealed that subjects developed stronger inter-regional connectivity between the primary and supplementary motor areas of the brain, which Ma et al. (2010) suggested, “may reflect long-term reorganization of the skilled motor network” (p. 64). Essentially, the brain created or strengthened neural connections as a result of these repetitive movements, which then served to increase the subjects' performance of the task.

This type of long-term reorganization is not only limited to motor tasks but can also be achieved by learning, as it relates to cognitive skills. In a study by Kühn et al. (2014), experimental subjects were trained to play a video game for at least 30 min a day over the course of 2 months. When compared with a control group, the game players brain scans showed an increase in gray matter in areas that effect cognitive skills such as spatial navigation, strategic planning, and working memory while also providing evidence of behavioral changes associated with navigation strategy (p. 1). These subjects not only learned how to play the game more efficiently but also displayed development in skills related to spatial navigation and strategic planning, which was evidenced by increased connectivity in areas of their brains corresponding with these skills.

In addition to neural restructuring related to task based learning and motor skill function, neuroplasticity can, and has, been utilized as a way to modulate thought and through thought, emotional responses. For example, neuropsychological research into the effectiveness of Cognitive Behavioral Therapy (CBT) has produced compelling results that speak to the ability of CBT to affect neural structures. CBT is a therapeutic modality based on modifying existing thought patterns by identifying these patterns and their corresponding emotional/behavioral responses, and working to help patients consider “more adaptive and accurate perspectives” (Beck Institute, 2016). In a randomized control trial utilizing CBT for social anxiety disorder, Goldin et al. (2014), found that patients undergoing a round of CBT evidenced improvements in “cognitive reappraisal-related prefrontal cortex responses (p. 1048).” These findings suggest that changes in neural structure related to the modulation of cognitive reappraisal-related brain responses, timing, and functional connectivity, serve as contributing factors in the effectiveness of CBT interventions (Goldin et al., 2014, p. 2).

Dharma Life Program builds on the current understanding and applications of neuroplasticity by implementing a methodology which integrates theories taken from previous utilizations of the concept of neuroplasticity (motor tasks, skill learning, and cognitive appraisal) and applies it in the context of a personality development program. This is achieved using three interventions: brain actions, mind actions, and real-world actions. Each of these interventions uses a different type of action to facilitate neural modification. Brain actions require that the participant play games that create new neural structures for the maladaptive trait. In mind actions, participants are encouraged to create new associations for situations that they have recently experienced that triggered the maladaptive trait. This results in modification of the neural structures associated with those situations. Real-world actions prompt the client to engage in real life activities that are typically either avoided by the individual or trigger an unwanted behavior, because of their maladaptive trait. The real-world actions create new associations/memories between the real world and new behaviors that counter the maladaptive trait to further modify neural structures.

To modify the maladaptive trait to become an adaptive trait, it is important to specify the actual change in the neural structure that needs to be accomplished. The Dharma Life program is based on the following three assumptions which enable the targeting of neural structures related to a specific maladaptive trait: (1) An individual can develop a maladaptive trait when they repeatedly experience external and internal stimuli that trigger maladaptive responses. (2) There is a commonality in the external and internal stimuli and the resulting decision-making process that creates the maladaptive trait. (3) This commonality creates patterns in both cognitive perceptions, thought processes, and behavior. The Dharma Life program defines this commonality as the “wiring statement.” Each type of maladaptive trait has a different wiring statement. Furthermore, to modify the maladaptive trait the Dharma Life program assumes that the user must be presented with the opposite of the trait's wiring statement and be given the opportunity to make decisions that reflect the acknowledgement and utilization of this opposite statement. The opposite of a trait wiring statement is a “rewiring statement.” In order to target the maladaptive trait and enable the participant to gain a more adaptive utilization of their trait, the individual has to perform a large number of actions/repetitions of the rewiring statement. The Dharma Life program relies on the Dharma Life smartphone application to aid the individual in carrying out a large number of actions/repetitions to modify the maladaptive trait.

The repetitions are implemented through three types of actions in the Dharma Life Application. The brain actions are games based on the rewiring statement that present fictitious situations in which the user must make decisions that are considered correct when they apply their rewiring statement to the situation. The mind actions enable the user to recall a past situation that they themselves have experienced and apply the rewiring statement to it. The user is then prompted to create alternative associations for that situation (by applying the rewiring statement) and consider alternative responses to that situation. The real-world actions allow the user to create new cognitive reappraisals for situations that either the user avoided or that triggered the incorrect behavior of the maladaptive trait. The premise of the Dharma Life program is that all the above actions modify the required neural structure to modify the maladaptive trait and allowing the user to transform it into an adaptive trait. In this preliminary study, we examine if Dharma Life Program enhances emotional intelligence.

### Hypothesis

**Figure 1:**
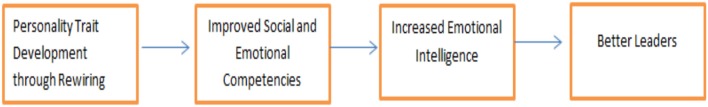
Conceptual map.

We anticipate that participation in the Dharma Life Program, through its targeting of maladaptive personality traits, will demonstrate a positive correlation in improving emotional intelligence. See Figure 1.

### The Dharma Life Program

#### The intervention

The Dharma Life Program aims to improve the maladaptive traits of individuals and can be used in various situation including leadership development. The intervention is carried out over a period of 8 weeks featuring two discovery sessions with a Dharma Life Sciences Mentor and 6 weeks of game play via the Dharma Life smart phone application, which is supported by a weekly 1 h session (via telephone call) with a Mentor. A trait is identified in the first two discovery sessions, after which the remaining six sessions introduce new “actions” each week for the client to engage with on the Dharma Life smart phone Application (App).

#### The Dharma Life App

The Dharma Life App is a smart phone application that is an integral component of the Dharma Life Program. The app gives the user trait specific access to “actions” which target the behaviors and thought processes of their identified trait. The application itself contains multiple types of “actions” which address specific aspects of the trait. The actions fall into the following categories:

#### Brain actions

**Figure 2:**
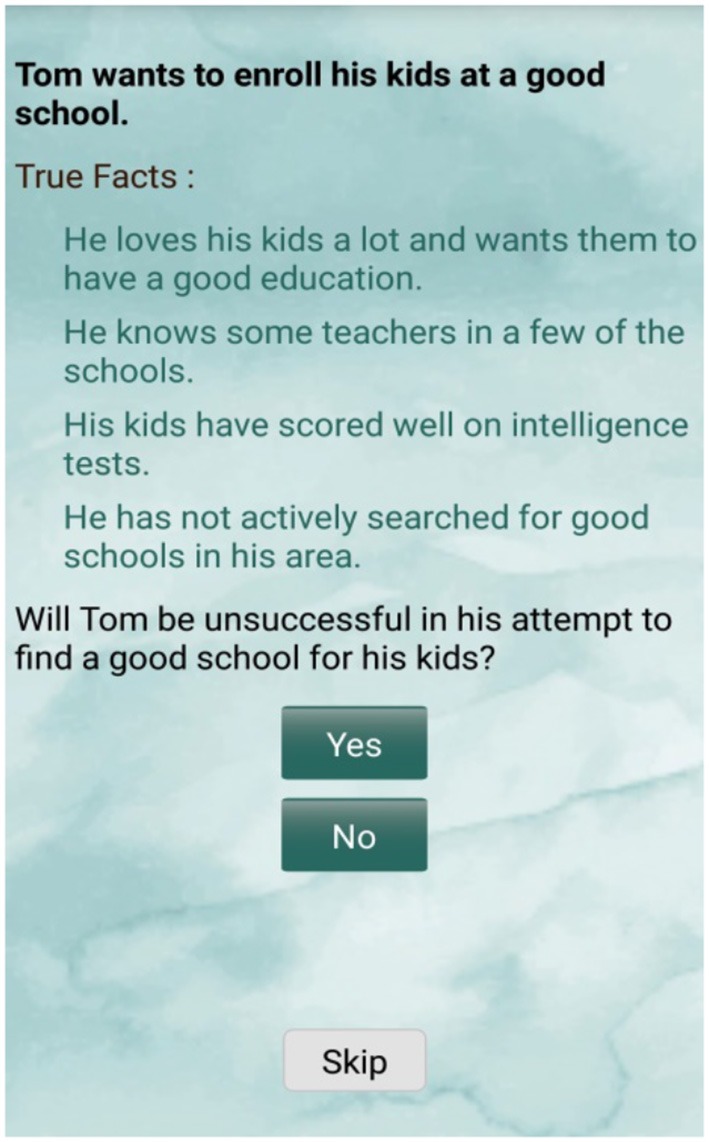
Fact-full game example.

These actions include games designed to target the underlying processes of one's trait. For example, the brain action, “fact-full,” includes a scenario accompanied by three to four facts. The participant is asked a question that corresponds to both the facts and the scenario. The facts are designed to cultivate an automatic response, contrary to the user's current trait, by instructing the user to select an answer based on a broader understanding of the situation. The user must answer based on the quantity of facts which suggest a certain response as opposed to allowing themselves to be guided by a single trait triggering fact. For equal measure, some answers do correspond with responses typical of an individual's trait. The reason being, the brain actions are aimed at helping the user; see the image below (Figure 2) is a screen shot from the game-Fact Full.

Here, there are more facts supporting a positive outcome, i.e., Tom will be successful in his attempt to find a good school for his kids.

#### Physical actions

These actions are used to balance the biochemistry associated with one's trait.

#### Mind actions

Mind actions reshape one's interpretation of the past by promoting a person to reflect on events that happened throughout his/her day and/or life. Moreover, the individual cultivates more informed conscious decision-making strategies through these actions. A few examples of such mind actions, journaling, alternative thinking, and other actions based on CBT.

#### Real-world actions

Real-world actions are based on exposure therapy by having the user proactively deal with situations that they tend to avoid and/or would trigger their trait. This could include observing people in one's day for specific behaviors that the individual aspires to emulate, etc. Some of these actions may be custom-created by the mentor and participant as well. For example, in the final weeks of the program, the participant is directed to brainstorm a live real-world action to carry out in their day that contradicts their normal behavior.

Additionally, the app displays a “trait scale” labeled with a zero on one end and a hundred on the other, as a means of giving the user a visual account of their progress throughout the program and provides the user with the means by which to share their progress with their mentor. To send the data associated with the users completed actions, the app contains a log report generator that assimilates one's progress into a single document. This report is sent to the mentor every week by the participant and provides the mentor with an overview of the individual's progress. This enables the participant and the mentor to discuss the actions the individual completed between sessions.

#### Dharma Life report analyzer

The mentor uses a report analyzer to review the participant's log report at the end of each week and to track their progress over the course of the program. The report analyzer compiles the data generated by the participant's log, which is sent to the Mentor before each weekly session, into a format that enable the mentor to view the details of their actions. For brain actions, the information would include where the client erred in their game play and see where they were most successful. The result of each weekly report dictates the discussion for the next session, following the first two discovery sessions.

## Methods

### Study design

In this study, participants underwent the Dharma Life Program as part of required coursework. Their emotional and social competencies was assessed before and after the program using the Emotional and Social Competency Inventory University Edition (ESCIU). This instrument was used to track changes in their emotional intelligence as a result of their participation in the Dharma Life Program. Descriptive and inferential statistical analyses were performed on the data to evaluate the effect of Dharma Life Program on emotional intelligence of the participants.

### Participants

Participants were 30 undergraduate students participating in a Leadership Development course at a university located in the mid-western United States. This was a self-selected group of students who were interested in development their leadership abilities. Of these students, 50% were majors in the sciences, technology, engineering, or mathematics (STEM) programs. Another 40% were business majors, and the remaining 10% were majors in fields related to the humanities or social sciences. The average age of the participants was twenty-one, with most of the students being in their senior year of their undergraduate education. Demographic data reflecting the gender or racial composition of the sample, was not available for analysis. Enrollment in the Dharma Life Program was offered as a means to satisfy a course requirement.

Of the 30 enrolled students, four did not complete the program and were excluded from the analysis. Two of these students dropped the class due to scheduling conflicts, one had a trait that had not yet been implemented in the Dharma Life Program, and the remaining student did not have access to a smart phone or comparable device.

### Measures

#### Emotional and social competency inventory, university edition (ESCIU)

The instrument used for assessing the emotional and social competencies of the participants was the ESCIU developed by Boyatzis (2007). This instrument assessed participant emotional and social competencies across five domains. However, for the purpose of our study, participants were only tested on four of the domains. The fifth domain, Cognitive Competencies was not used because “systems thinking” and “pattern recognition” were not expected to change by changing personality traits through the Dharma Life Program.

The four domains the participants were scored in were: Self-Awareness, Self-Management, Social-Awareness, and Relationship Management. Each of these domains has corresponding competencies. Students were scored in Emotional Self-Awareness (Self-Awareness), Empathy and Organizational Awareness (Social Awareness), Achievement Orientation, Adaptability, Emotional Self-Control, Positive Outlook (Self-Management), and Conflict Management, Coach and Mentor, Influence, Inspirational Leadership, and Teamwork (Relationship Management) (Boyatzis, 2007).

Students were given the ESCI both before beginning the Dharma Life Program and after completing the Dharma Life Program. These scores served as the primary source of data for this preliminary study.

#### Client trait questionnaires

Dharma Life trait questionnaires were developed to inquire as to the frequency and severity of trait behaviors. The questionnaires used were previously validated measures which spoke to trait behaviors (see Appendices A and B in Supplementary Material for a list of traits and a list of validated questionnaires).

Editable PDFs were sent to participants to fill out. The questionnaires were scored out of 100. The questionnaires were used to confirm that the students did have the trait behaviors that they expressed verbally in session 1 and the frequency and severity of these behaviors. The Mentor chose the trait to be worked on by asking the student to reflect which behaviors came first in their life. This trait was assumed to be the most deeply-rooted and the one that needed to be worked on first.

### Procedure

#### Informed consent and ESCI pre-assessment

Before the study could begin, participants were given an informed consent form, detailing the scope of the study, and ensuring confidentiality, to sign. In addition to explaining the scope of the study and that client information is kept confidential, the informed consent form notified the participants that the Dharma Life Program is not a mental health or medical service. Along with the informed consent forms to Dharma Life Sciences personnel, the participants were directed to complete an ESCI assessment to serve as the pre-test measure. The pre-ESCI was given to the students to evaluate their emotional and social intelligence at baseline (i.e., before the Dharma Life Program). Student's completed the ESCI using paper forms procured from the vendor. The assessment was administered by their leadership professor. The students were provided with detailed instructions to calculate their scores. They submitted these scores and their reflection on it as Pre-Dharma Life Self-Assessment Assignment. Following completion of the ESCI assessment, students were paired with a Dharma Life Mentor. Qualifications for Dharma Life Mentors, included having at least a Master's degree from an accredited institution, in either clinical or counseling psychology. The Mentor and the student decided upon a day of the week and a time for their first session via email exchange. This decision (day/time) would serve as the scheduled session time for the duration of the study, barring unforeseen circumstances. For each session, the Mentor called the client. All sessions were conducted via telephone call.

#### Discovery, weeks 1 and 2

The first 2 weeks of the Dharma Life Program were “Discovery” weeks, during which participants had two sessions where they spoke on the phone with a Dharma Life Mentor. The sessions were each scheduled 1 week apart.

##### Week 1

The first session is conducted in a manner similar to a phenomenological inquiry, insofar as the participant is asked an open-ended question (“If you had a magic wish, which of your behaviors would you change?”) and directed to reply candidly. Following the participant's response, the Mentor will ask follow-up questions until a maladaptive trait, based on repetitious behavior, and thought patterns, emerges. Our definition of “maladaptive” in this context means either having too much or too little of the trait manifestation, which has subsequently negatively affected the individual's effectiveness and growth. Following the end of this session, the Mentor sent the client a few questionnaires that correspond to trait/traits that the client might potentially possess. The Mentor chooses which questionnaires to send to the student as a result of maladaptive trait behaviors or thought patterns indicated by the client during this session.

##### Week 2

The second session serves as an opportunity for the Mentor and the participant to reflect on the previous week and confirm the client's trait. The discussion during this session was guided by the results of the trait questionnaires that the Mentor sent via email to the client after the first session. These questionnaires were scored by the Mentor prior to the start of the session. The session began with the Mentor calling the client at the scheduled time. The participant was asked to reflect on the first session and the goals they hope to achieve if they could change the “wished away” behavior. The mentor then discussed the results of the questionnaires with the client and explains that the questionnaires sought to identify trait related behaviors and thought processes which the client engaged in most frequently. The Mentor then asked the client which trait the client remembers as affecting them first in their life, in case of multiple traits identified. Once identified by the client as the first trait, the mentor explained that this first trait will be the trait that the program will work on. Upon identifying this trait, the Mentor told the participant the wiring statements associated with the trait and engaged in a discussion with the client about the trait. Topics for discussion included but were not limited to: the characteristics of the client's trait, how the trait is limiting the client, and how the client feels they would be different if not as strongly influenced by their trait. As with all sessions, this session lasted for ~1 h. At the close of the session, the Mentor asked the client to reflect on the discussion and scheduled the next appointment.

#### Dharma Life trait program: week 3–week 8

##### Week 3

This session began with the Mentor requesting that the client to download the Dharma Life Science smart phone application. Once the application had been installed, the mentor guided the client through the app's features. The client was directed to review the “info” section, which offered a vignette detailing information about the trait, as well as the “habits” section which granted the participant access to the actions. The participant was then instructed to review the information in the “info” tab and to play one session each of all the brain actions unlocked, to familiarize themselves with the games. Then they were asked to begin playing the brain actions over the course of the next week. These brain actions, the client was informed, would serve as the topic of discussion in the next session. The client was then guided through setting up the log report and how to send the report to the Mentor. The Mentor informed the client that they should send a log report to them the day before their next session.

Upon finishing the app set-up and installation the mentor explained the reasoning for the actions/games the client will play. Then, the mentor provided and explained the wiring statement. The mentor reiterated that an individual obtains this wiring through repetitive experiences. The mentor presented the wiring in relation to the participant's specific behaviors/thoughts.

To end the session, the Mentor instructed the client to begin playing the actions which were unlocked for them in each category—Brain, Physical, mind, and real world. The participant was reminded to send the log report to the mentor and to play the indicated games for at least 15 min a day.

##### Week 4

In the interval between sessions the client completed “Brain Actions” and “Physical Actions” and sent the log of their game play to the Mentor for their review. The Mentor loaded the log into the Report Analyzer and reviewed the client's actions before calling them for the session. Next, the participant and mentor reviewed the results of the brain actions. The mentor and the participant discussed any errors that the participant made and went over how the participant's wiring lead him/her to the wrong choices on the brain games. The mentor then pointed out how the rewiring statement can guide the student in making better choices for himself/herself. Moreover, the mentor connected the student's thought processes to his/her goals and helped guide the participant to identify ways in which they might be able to apply the rewiring statement to help achieve their goals. The participant was told what cognitive distortions accompany his/her trait and how they interfere with his/her ability to see his/her life more clearly. And they were connected to the wiring statements. This made it easy for the participant to understand and reflect, which enhances the rewiring process. At the end of this session, the mentor instructs the participant to being playing the next set of actions. “Mind Actions” as well as the “Brain Actions.”

##### Week 5

In between the week 4 session and the week 5 session, the participant completed “Mind Actions” and sent their log report to their Mentor. As in week 4, the client was asked to reflect on actions they have completed on the app over during the week. The Mentor and the client and went over any incorrect answers. Once the review has been completed, the Mentor introduces rewiring statements to the clients, so that they start applying them in everyday scenario and change the rigid pattern that they have formed for themselves. Then the mentors requested the client to begin working on the “Real-world Actions, 1 & 2” during the upcoming week. The Mentor ended the session by reminding the client to play on Brain, physical and mind actions and to send them their log report the day before the next session so that the Mentor can review and analyze the client's app usage.

##### Week 6

The mentor began the week 6 session by asking the client about their experiences with their real-world action 1 & 2. The mentor follows up this discussion with an explanation of reasoning behind the real-world actions and works with the client to identify where they applied their trait wiring and how they might be able to apply the rewiring statement instead in the future. The Mentor and the student then brain stormed the next level of real world actions (real world actions level 3) in line with the student's identified goals. Together they decided what actions the student should attempt in the interval between session. The student was prompted to consider ways in which applying the re-wiring statement would help them complete the real-world action and how it could further their goals. As with all sessions, the student was reminded to play rest of the actions along with real world action and send the mentor their log report prior to the following session.

##### Week 7

At the beginning of the week 7 session, the mentor and the student discussed the real-world actions (real world actions level 3) that the student took in the interval between sessions, and also the other activities, if there were any errors. The student was prompted to discuss their feelings surrounding undertaking the real-world action and their relative success in completing the action. Most of the discussion was based on these insights. The Mentor drew the student's attention to incidents where they displayed behaviors related to their trait and where they applied both their wiring and re-wiring statements. The student was asked, during the discussion, to reflect on how they could have applied the rewiring statements more efficiently in areas where they fell into the pattern of the wiring statement. Toward the end of the session the mentor and the student brain stormed further real-world actions (real world actions level 4), this time following the guidelines of real world actions 4. To undertake real world actions level 4, the student and the mentor designed an action for the student that poses a direct challenge to his/her trait, as opposed to discussing actions that the student identified as having triggered their trait in the interval between sessions, as they had in session 6. Once the student's actions had been agreed upon and the client had been reminded to send their log report, the session concluded.

##### Week 8: real-world actions 4 and relapse prevention

The Mentor began the last session of the program, by asking the student about their experience carrying out their assigned real-world actions. The client was then instructed to reflect on their experiences with the program and their wiring and re-wiring statements. The Mentor discussed relapse prevention with the client. The discussion of relapse prevention involved the mentor prompting the client to review their trait triggers followed by the mentor having the client discuss how they would combat the triggers utilizing the rewiring statement that they have learned. The Mentor then notes that the client can spend 15 min a week on the app to continue their progress or if they feel that they are falling back into old routines. Additionally, the final Real-World Action (real world action level 5) is discussed. Real World Action level 5 involves the client becoming a mentor to someone else with their specific trait. The student was prompted to guide this person through the skills that the student themselves developed over the course of the program. Finally, the mentor ended the session by congratulating the client on completing the Dharma Life Program. And the mentor sent the same trait questionnaire which was sent in initially, for the post-test analysis.

#### ESCI post-assessment

Following the week 8 session, the ESCI was again administered to the students by their professor. As with the ESCI administered prior to beginning the Dharma Life Program, the post-Dharma Life Program ESCI was completed using a paper test that was then graded by the students.

### Data analysis

Pre- and Post-ESCI Self-Assessment scores were analyzed in two steps.

#### Descriptive statistics

Percentage improvements were calculated for each of the domains and competencies of the ESCI scale. These improvements were calculated using:

$$\begin{array}{l} \frac{\text{Posttest}-\text{Pretest}}{\text{Pre Test}}*\;100 \end{array}$$

#### Inferential statistics

Given our that our data came from a relatively small sample of pre- and post-test scores from the same population, utilizing paired sample statistical analytic measures was appropriate. Data sets investigated were the overall pre- and post-ESCI reports, the pre-test and post-test changes across each domain evaluated by the ESCI and each participant's three lowest competencies.

A paired sample *t*-Test was used to examine if there was a significant difference between the pre-test and post-test data gathered from the ESCI evaluations. The Pearson's Correlation was used to determine the relationship between the pre-test and post-test means. These measures were used to investigate the effect size across the overall pre-test and post-test ESCI results, the pre-test and post-test results for each individual ESCI domain, and pre-test and post-test changes in each participant's three lowest competencies.

For the analyses, the significance level was set at *p* = 0.01. Statistical analysis was performed using a Microsoft Excel plug in and recorded using Microsoft Excel.

## Results

To test the significance of the relationship between the ESCI and the Dharma Life program, a paired samples one-tailed *t*-test was conducted for the self-reported ESCI scores before and after the program's completion. We assumed a normal distribution for these scores.

Descriptive statistics and *t*-test results for the four overall domains combined and individually showed a significant difference in the same direction for nearly all analyses (Table 1).

**Table 1:** Results of *t*-tests and descriptive statistics of effect of dharma life program on domains of emotional intelligence.

	**Pre-test**		**Post-test**		**Difference**	**T**
	***X***	***SD***	***X***	***SD***		
Combined domains	221.58	22.81	238.27	19.98	−16.69	−3.78^*^
Self-awareness	18.23	3.71	19.58	2.45	−1.35	−2.60^*^
Social awareness	39.46	4.84	40.62	4.27	−1.15	−1.46
Self-management	73.92	10.46	79.42	7.56	−5.50	−3.37^*^
Relationship management	89.96	9.74	97.65	10.12	−7.69	−4.15^*^

* *p < 0.01*.

The combined domain mean pre-test score was 221.59 (*SD* = 22.81) and the mean post-test score was 238.27 (*SD* = 19.98). Social awareness was the only individual domain to not have a significant difference in ESCI scores with a pre-test mean of 39.46 (*SD* = 4.84) and a post-test mean of 40.62 (*SD* = 4.27).

Descriptive statistics and *t*-test results from the lowest ESCI competencies showed a significant difference in the same direction for all the analyses (Table 2).

**Table 2:** Results of *t*-tests and descriptive statistics of effect of dharma life program on lowest ESCI competencies.

	**Pre-test**		**Post-test**		**Difference**	**T**
	***X***	***SD***	***X***	***SD***		
Lowest competency	13.42	2.21	17.42	2.18	−4.00	−8.74^*^
Lowest two competencies	28.42	4.27	35.12	4.41	−6.69	−7.09^*^
Lowest three competencies	44.77	5.92	53.73	5.72	−8.96	−6.98^*^

* *p < 0.01*.

Notably, the lowest competency ESCI scores for participants had a mean pre-test score of 13.42 (*SD* = 2.21) and a mean post-test score of 17.42 (*SD* = 2.18).

## Discussion

This study investigated the effect of participation in the Dharma Life Program on the ESCIU scores. We found support for our hypothesis, that completion of the Dharma Life Program improves ESCI scores. Using an alpha value of 0.01, we found a statistically significant positive change between the pre-test and post-test scores for almost all domains. This study provides preliminary results that address a novel approach to improving Emotional and Social Intelligence. The Dharma Life Program targets maladaptive traits, which affects situational perceptions, emotional responses, and behaviors, including those measured by the ESCI (e.g., Conflict Management, Emotional Self-Control, Teamwork, etc.).

While our results showed a statistically significant improvement in most domains, there was not a significant improvement in the domain “Social Awareness.” A potential reason for this is that the most commonly found maladaptive trait in this sample was “High Empathy.” The Dharma Life Program would have targeted this trait in such a way as to reduce, rather than enhance empathy. Empathy, however, is one of two competencies investigated in the Social Awareness domain, where the assumption seems to be that empathy should be enhanced. Therefore, it may be the case that the success of the Dharma Life Program in assisting people with a maladaptive employment of empathy would lead to results contrary to our hypothesis in this instance. We also suspect that empathy as a cognitive skill brings it with the ability to set boundaries (Poonamallee and Goltz, 2014) which is essential for leadership effectiveness while high empathy as a maladaptive personality trait could affect the leader negatively. This poses a potentially interesting question for future research in which empathy as a cognitive competency can be compared with empathy as a personality trait and the dynamics understood better.

This paper makes a unique contribution to individual and organizational development practice and research. Emotional intelligence is considered to be the most reliable predictor for leadership effectiveness and professional success in several contexts and several professional disciplines including even the most cognitively oriented ones such as engineering. Many academic institutions, public institutions and corporations understand the relevance of emotional intelligence and have begun to invest heavily in developing it among its students, members, and employees, respectively. However, developmental interventions have been more focused on creating “aha” moments or epiphanies through offsite and other developmental workshops, reliable tools and interventions for actual development of EI are mostly limited to executive coaching. Our findings suggest that the Dharma Life Program, through its targeting of maladaptive personality traits, had a positive impact on ESCI scores. These findings may indicate that the Emotional Intelligence of our sample did improve as a result of participation in the Dharma Life Program. We argue that this could illustrate that, programs like the Dharma Life Program, which seeks to address impediments to success caused by behavior and thought processes associated with a maladaptive personality trait, may prove effective in improving Emotional Intelligence. Given that the link between a leadership capabilities and scores on the ESCI assessment have been correlated (Boyatzis et al., 2012), further research utilizing the Dharma Life program could prove to be a vital avenue to improve leadership development programs.

A common pitfall of many leadership programs is their inability to address attitudinal and behavioral issues across different levels of an organization (Beer et al., 2016). However, the Dharma Life Program avoids this concern because it does in fact, focus on attitudinal and behavioral issues at an individual level. As a result, the Dharma Life Program potentially serves as a more universally applicable tool for leadership development. Therefore, it may be worthwhile to continue investigating the effect of the Dharma Life program on ESCI and other leadership assessments. Future studies should continue to explore the degree to which Dharma Life and ESCI are related as well as incorporate 360 assessments in leadership and management scenarios.

Results of this study are encouraging and offers a novel and innovative science-based intervention for developing emotional intelligence competencies. This could be used in university settings for student success and student leadership, organizational settings for leadership development and by individuals for personal development to overcome the challenges of their maladapted traits. Unlike most interventions that either span a very long time such as therapy interventions or very short periods such as workshops and lectures, Dharma Life program offers a manageable timeframe that results in effective enhancement of emotional intelligence in individuals. Prior to our investigation, ESCI scores were typically addressed in studies which utilized coaching as the mechanism for change. However, results of this study suggest that there may be other effective means by which to improve these competencies. This study provides support for future research initiatives which seek to improve emotional and social competencies through alternative methodologies.

Extrapolating from the assumptions of Dharma Life Program and findings from this study, we can surmise that personality traits also shape other key competencies such as creativity and innovation that are crucial for solving major problems such as climate change, global health etc. In fact, the most contemporary approaches to innovation such as Design Thinking place empathy at the center of the process. However, much like leadership development, even though there is an increasing understanding of what one needs to be more creative or innovative, tools to enhance creativity and innovation are limited to a few such as art and theater based approaches and improvisation style exercises. While these are useful to spark curiosity and interest, there is no evidence that these approaches result in modified neural structures that allow individuals to make new connections that are essential for creativity and innovation. Dharma Life Program can be used to identify maladaptive traits that can block creativity and innovation and develop adaptive traits that will encourage and nurture them. This is another area for potential application and research in ecologically valid real-world contexts. Further research in establishing connections between Dharma Life Program traits and specific competencies that may be deemed to be valuable by an organization can help develop customized organizational development programs that will help shift organizational cultures through interventions at the individual levels.

## Limitations

Despite the fact that our findings largely support the hypothesis that participation in Dharma Life Program results in an increase in emotional intelligence, the study is not without its limitations. First, as a consequence of our small sample size and lack of demographic data, it would be premature to assume that these findings would be applicable to a larger population. A larger sample may provide means by which to assume a measure of generalizability.

Furthermore, this study did not include a control group, which subsequently limited our ability to calculate other statistical analyses. As a result, it is difficult to determine whether the increase in the scores in the majority of ESCI domains is wholly attributable to the impact of the Dharma Life Program. Since the experimental sample consisted entirely of students undertaking a leadership and development course, it is possible that other assignments may have contributed to improvements in their ESCI scores.

Additionally, our data analysis was conducted solely through the ESCI self-assessment measure. The issue inherent in self-assessment is that it does require a level of self-awareness that remains somewhat stable both pre- and post-test. It could be the case that in some instances, scores were not as reflective of a true measure of improvement, as some people may have initially evaluated themselves unfairly.

## Ethics statement

The study was carried out in accordance with the recommendations of Michigan Technological University Institutional Review Board with written informed consent from all subjects. All subjects gave written consent in accordance with the Declaration of Helsinki. The protocol was approved by Michigan Technological University Institutional Review Board.

## Author contributions

LP provided direction, framing, and conducted the quasi-experiment in her class and led the writing of the paper. AH framed and led the revision. MN contributed to the writing of the initial submission. AM conducted the statistical analyses and reviewed the paper.

### Conflict of interest statement

The second author, MN was employed by company Dharma Life when the study was conducted. Currently she is not affiliated them. All other authors declare that the research was conducted in the absence of any commercial or financial relationships that could be construed as a potential conflict of interest.

## Footnotes

^1^https://www.merriam-webster.com/dictionary/personality
