# Supplementary material for: Improving Emotional Intelligence through Personality Development: The Effect of the Smart Phone Application based Dharma Life Program on Emotional Intelligence
Source: Front Psychol. 2018 Feb 23;9:169. doi: 10.3389/fpsyg.2018.00169 (PMC5829461; doi:10.3389/fpsyg.2018.00169)
Supplement: Supplementary file 1 [file Table1.docx]

Appendix A: **Dharma Life Maladaptive Traits Table**

| Anger | Acceptance | Depression | Elation | Worry | Certainty | Anxiety | Certainty |
| --- | --- | --- | --- | --- | --- | --- | --- |
| Social Anxiety | Social Certainty | Low Self-Control | Inhibition | Low Self-Confidence | Superiority | High Empathy | Low Empathy |
| Pessimism | Optimism | Low Trust | High Trust | Submissiveness | Rebelliousness | Impatience | Passivity |
| Sensitivity | Guarded | Envy | Content | Perfectionism | Sloppiness | Laziness | Obsessiveness |

Appendix B: **Scales and Scoring Guide**

| **Trait and the form code** | **Name of the Scale in** | **Scoring** | **Cut off range** |
| --- | --- | --- | --- |
| F-3 Anger | Clinical Anger Scale | Normal Scores -  A=0, B=1, C=2, and D=3. | The minimum score is 0 and the maximum score is 63.  The range is:  0-13 = Minimal Clinical Anger  14-19 = Mild Clinical Anger  20-28 = Moderate Clinical Anger 29-63 = Severe Clinical Anger |
| F-4 Depression | Becks Depression Inventory | A value of 0 to 3 is assigned for each answer and then the total score is obtained. | The minimum score is 0 and the maximum score is 63.  The range is:  0-10 = These ups and downs are considered normal  11-16= Mild mood disturbance  17-20= Borderline clinical depression  21-30= Moderate depression  31-40= Severe depression  Over 40= Extreme depression |
| F-5 Worry | Penn State Worry Questionnaire | Normal Scores -  1=5, 2=4, 3=3, 4=2, 5=1.  Reverse Scores are obtained for Items 1, 3, 8, 10, and 11 1=1, 2=2, 3=3, 4=4, 5=5  Sums are obtained | The minimum score is 16 and the maximum score is 80.  The range is:  16-39 = Low Worry  40-59 = Moderate Worry  60-80 = High Worry |
| F-6 Anxiety | Beck’s Anxiety Scale | A value of 0 to 3 is assigned for each answer and then the total score is obtained. | The minimum score is 0 and the maximum score is 63.  The range is:  0-7 = Minimal Anxiety  8-15 = Mild Anxiety  16-25 = Moderate Anxiety  26-63= Severe Anxiety |
| F-7 Social Anxiety | Social Phobia Inventory (SPIN) | Normal Scores -  0=0, 1=1, 2=2, 3=3, 4=4  No reverse scoring | The minimum score is 0 and the maximum score is 68.  The range is: Less than 20= none  21-30 = Mild  31-40= Moderate  41 – 50= Severe  51 or more =Very Severe |
| F-8 Self- Control | Brief Self Control Scale | Normal Scores -  a=5, b=4, c=3, d=2, e=1.  Reverse Scores are obtained for Items 4,5 and 6  a=1, b=2, c=3, d=4, e=5  Add up all the points and divide by 10. | The maximum score on  this scale is 5 (extremely self controlled), and the lowest scale on this scale is 1 (not at all self-controlled)  Higher scores reflects more self-control. |
| F-9 Self Confidence | Rosenberg Self Esteem Scale | Normal Scores -  Items 1, 3, 4, 7, and 10 SA=3, A=2, D=1, and SD=0  Reverse Scores are obtained for Items 2, 5, 6, 8, and 9 score SA=0, A=1, D=2, and SD=3 | The minimum score is 0 and the maximum score is 30.  The range is:  0-15 = low  15 and 25 = average  25-30 = high |
| F-10 Empathy | Toronto Empathy Scale | Normal Scores -  Positive Items 1, 3, 5, 6, 8, 9, 13, 16.  0= 0, 1=1, 2=2, 3=3, 4=4.  Reverse Scores are obtained for Negative Items 2, 4, 7, 10, 11, 12, 14, 15.  0= 4, 1=3, 2=2, 3=1, 4=0.  Scores are summed to derive total score. | The minimum score is 0 and the maximum score is 64.  Higher the score , higher the empathy level |
| F-11 Pessimism | Beck’s Hopelessness Scale | Count one point if the items are answered as following:   1. False 2. True 3. False 4. True 5. False 6. False 7. True 8. False 9. True 10. False 11. True 12. True 13. False 14. True 15. False 16. True 17. True 18. True 19. False 20. True | The minimum score is 0 and the maximum score is 20.  The range is:  0-3 = Minimal  4-8 = Mild  9-14 = Moderate  15-20 = Severe |
| F-12 Trust | General Trust Scale | The score for each item is averaged together to form a measure of generalized trust.  SD=1, D=2, N=3, A=4, SA=5 | The minimum score is 6, and the maximum score is 30.  Higher the score higher the trust. Item analysis has to be done . |
| F-13  Submissiveness | Submissive Behaviour Scale | Add up all the items.  N=0, R=1, S=2, M=3, A=4 | The minimum score is 0 and the maximum score is 64.  The range is:  0-20 – Low Submissiveness  21-40 – Average Submissiveness  41-64 – High submissiveness |
| F-15 Self-Control | Baratt Impulsiveness Scale | First Order factors and their items  Attention: 5, 9*, 11, 20*, 28  Motor: 2, 3, 4, 17, 19, 22, 25  Self-Control: 1*, 7*, 8*, 12*, 13*, 14  Cognitive Complexity: 10*, 15*, 18, 27, 29*  Perseverance: 16, 21, 23, 30*  Cognitive Instability: 6, 24, 26  Items with * mark are reverse scored. |  |
| F-16 Sensitivity | Highly Sensitive Person Scale | If more than fourteen of the questions are answered as true of yourself, it is evaluated as probably highly sensitive. | Item analysis has to be done. The minimum score is 0 and the maximum score is 27.  0-13 = Average  14-27 = Highly Sensitive. |
| F-18 Envy | Dis-positional Envy Scale | Items are scored as  SD=1, D=2, N=3, A=4, SA=5  No reverse scoring.  Add up the numbers placed in front of the items. | The minimum score is 8 and the maximum score is 40.  Higher scores reflect a greater tendency to experience envy. |
| F-19 Perfectionism Scale | Multidimensional perfectionism Scale | No reverse scoring.SD=1, D=2, N=3, A=4, SA=5 | The minimum score is 35 and the maximum score is 175.  Higher scores indicates higher perfectionism levels. |
| F-20 Scale to Laziness | Procrastination Scale | The items are scored by summing the item responses on a scale from 1 to 4  “That's me for sure”= 4,  “That's my tendency”=3,  “That's not my tendency”=2  “That's not me for sure”=1  Items 6, 8, 13, 17, 25, 27, 29, 30, 33, 34 are reverse-scored.  “That's me for sure”= 1  “That's my tendency”=2  “That's not my tendency”=3  “That's not me for sure”=4 | The minimum score is 35 and the maximum score is 140.  Higher scores equalling greater tendencies to procrastination |
